# Screening of Bioactive Compounds in the Tianma–Gouteng Pair and Its Protective Effects on an L-NAME-Induced Trophoblast Organoid Injury Model

**DOI:** 10.3390/ph19101540

**Published:** 2026-09-28

**Authors:** Aolin Zhang, Baofeng Rao, Yu Lu, Ying Liu, Anqi Zheng, Ruipeng Liu, Xinyue Liu, Tao He, Chi Chiu Wang, Lu Li

**Affiliations:** 1 Pharmaceutical Informatics Institute, School of Pharmacy, Zhejiang University, Hangzhou 310058, China; 22119174@zju.edu.cn (A.Z.); bf_rao@163.com (B.R.);; 2 State Key Laboratory of Chinese Medicine Modernization, Innovation Center of Yangtze River Delta, Zhejiang University, Jiaxing 314100, China; 3 Department of Obstetrics and Gynecology, The Chinese University of Hong Kong, Shatin 999077, Hong Kong; 4 International Science and Technology Cooperation Base of Chinese Medicine Modernization and Big Health, Innovation Center of Yangtze River Delta, Zhejiang University, Jiaxing 314100, China; 5 Modern Chinese Medicine and Reproductive Health Joint Innovation Center, Innovation Center of Yangtze River Delta, Zhejiang University, Jiaxing 314100, China

**Keywords:** Tianma–Gouteng pair, bioactive compound, preeclampsia, transcriptome sequencing, trophoblast organoids

## Abstract

**Background:** Preeclampsia (PE) is a hypertensive, high incidence disorder occurring only during pregnancy and has high incidence. The Tianma–Gouteng pair (TGP) is used in traditional Chinese medicine for cardiovascular indications, but its candidate bioactive constituents and relevance to PE remain unclear. **Objectives:** This study aimed to identify candidate TGP compounds relevant to PE and elucidate their effects in experimental trophoblast models. **Methods:** Placental transcriptomes and network pharmacology analysis were used as an exploratory discovery stage. Candidate compounds of TGP were then evaluated using ADRA1A-overexpression cells and trophoblast organoids, molecular docking, surface plasmon resonance (SPR), and an L-NAME-induced trophoblast organoid injury model. **Results:** Placental injury and dysfunction were observed in PE mice. Integrated analyses prioritized α1A-adrenergic receptor (ADRA1A) as a candidate TGP-associated target. Five database-derived compounds of TGP were evaluated experimentally. Isorhynchophylline (SRH) showed direct binding to ADRA1A by SPR (equilibrium KD = 4.67 μM). In trophoblast organoids, 15 μM SRH did not significantly reduce viability and was associated with partial restoration of Ki67 and HLA-G expression after L-NAME injury. **Conclusions:** The data supported ADRA1A as a prioritized candidate target and SRH as a candidate compound with ADRA1A binding and activity in the experimental models. However, the current study did not establish that the biological effects of SRH depend on ADRA1A or demonstrate clinical efficacy of TGP or SRH in PE.

## 1. Introduction

Preeclampsia (PE) is a pregnancy-related hypertensive disorder that is characterized by new-onset hypertension and proteinuria after 20 weeks of gestation and often accompanied by multiorgan dysfunction [1]. It complicates approximately 2–8% of pregnancies worldwide, representing a leading cause of maternal and perinatal morbidity and mortality [2]. Despite extensive research, its exact etiology remains incompletely understood, and current clinical management primarily focuses on symptomatic control and timely delivery, as no effective pharmacological intervention can halt disease progression without compromising fetal safety [3].

The placenta plays a central role in the pathogenesis of PE [4]. Inadequate trophoblast invasion and impaired remodeling of uterine spiral arteries lead to placental hypoxia, oxidative stress, and release of anti-angiogenic factors, such as soluble fms-like tyrosine kinase 1 (sFlt-1), which together contribute to maternal systemic inflammatory response and endothelial injury [5,6]. Recent advances in placental research, including the development of trophoblast organoid models, have provided new opportunities to study PE pathophysiology and evaluate potential therapeutic agents in a physiologically relevant context [7]. This includes our recently developed N^G^-nitro-L-arginine methyl ester (L-NAME)-induced trophoblast organoid injury model (patent application CN202511878852.5; under substantive examination), which we used in this study.

Adrenergic receptors are cell surface glycoproteins belonging to the G protein-coupled receptor superfamily that recognize and selectively bind to the catecholamines norepinephrine and epinephrine that are released from sympathetic nerve endings and the adrenal medulla [8]. These receptors play integral roles at the interface between the sympathetic nervous and cardiovascular systems, with critical functions in rapidly regulating cardiac and vascular functions [9,10]. Adrenergic receptors are classified into α1, α2, and β subtypes, each with multiple subfamilies, and their distinct functions are mediated through different intracellular signaling pathways [11,12,13]. Adrenergic receptors are common therapeutic targets for anti-hypertension drugs. According to different selectivity of receptor subtypes, they are mainly classified into three categories: (1) β-adrenergic receptor antagonists, such as bisoprolol and metoprolol [14]; (2) α1-adrenergic receptor antagonists, such as prazosin and terazosin [15]; and (3) dual αβ receptor blockers such as carvedilol and labetalol [16,17].

Studies have revealed the potential of adrenergic receptors as therapeutic targets for PE. In human placental vessels, α1A receptors, along with α1B and β2 receptors, are considered important subtypes involved in regulating contractility [18]. A recent study proved that adrenergic α2 receptors regulated the expression of syncytin-1 and -2, as well as that of β-human chorionic gonadotropin, thus influencing cytoplasmatic fusion and the migration of trophoblast cells, which led to intrauterine growth restriction and severe PE [19]. Adrenergic receptor α1A (ADRA1A) is also implicated in the development of hypertension during pregnancy. In pregnancy-associated hypertensive mouse models, the mRNA levels of *Adra1a* in the heart were significantly reduced under hypertensive conditions, and changes in *Adra1a* expression were involved in progressive cardiac hypertrophy [20].

Traditional Chinese medicine (TCM) has long been used in the clinical management of pregnancy-related disorders [21,22], including PE [23,24], with beneficial effects in improving maternal and fetal outcomes. Among various TCM formulations, the Tianma–Gouteng pair (TGP), comprising *Gastrodia elata* (*Rhizoma Gastrodiae*, Tianma) and *Uncaria rhynchophylla* (*Ramulus Uncariae Cum Uncis*, Gouteng), is a well-known herbal combination that is traditionally used to treat hypertension, dizziness, and convulsions [25,26]. Clinical practices have confirmed the therapeutic effects of TGP as adjunct therapy for hypertensive disorders during pregnancy [27]. Recent studies have elucidated pharmacological mechanisms underlying protective effects of TGP against PE. Tianma–Gouteng Decoction regulates oxidative stress and nitric oxide synthetase in human extra-villous trophoblasts [28]. Rhynchophylla alkaloids derived from *U. rhynchophylla* and gastrodin derived from *G. elata* synergistically lowered blood pressure and reduced blood pressure variability in spontaneously hypertensive rats [29,30]. Moreover, a study has shown that corynoxeine, isorhynchophylline, isocorynoxeine and rhynchophylline from *U. rhynchophylla* bound to ADRA1A in prostate cells [31]. However, the specific bioactive compounds in TGP responsible for these effects and underlying molecular mechanisms remain largely unexplored.

In this study, we aimed to identify candidate targets in PE placentae and candidate bioactive compounds from TGP. We hypothesized that selected bioactive compounds from TGP protect trophoblast function by modulating ADRA1A related signaling. The transcriptomic and network analyses were used in an exploratory discovery stage, whereas compound screening, binding assays, cell experiments, and trophoblast organoid experiments were used in a validation stage. Because receptor dependence was not directly tested, this design evaluated consistency with an ADRA1A-related mechanism rather than establishing causality.

## 2. Results

### 2.1. Placental Injury and Dysfunction in PE Mice

**Figure 1:**
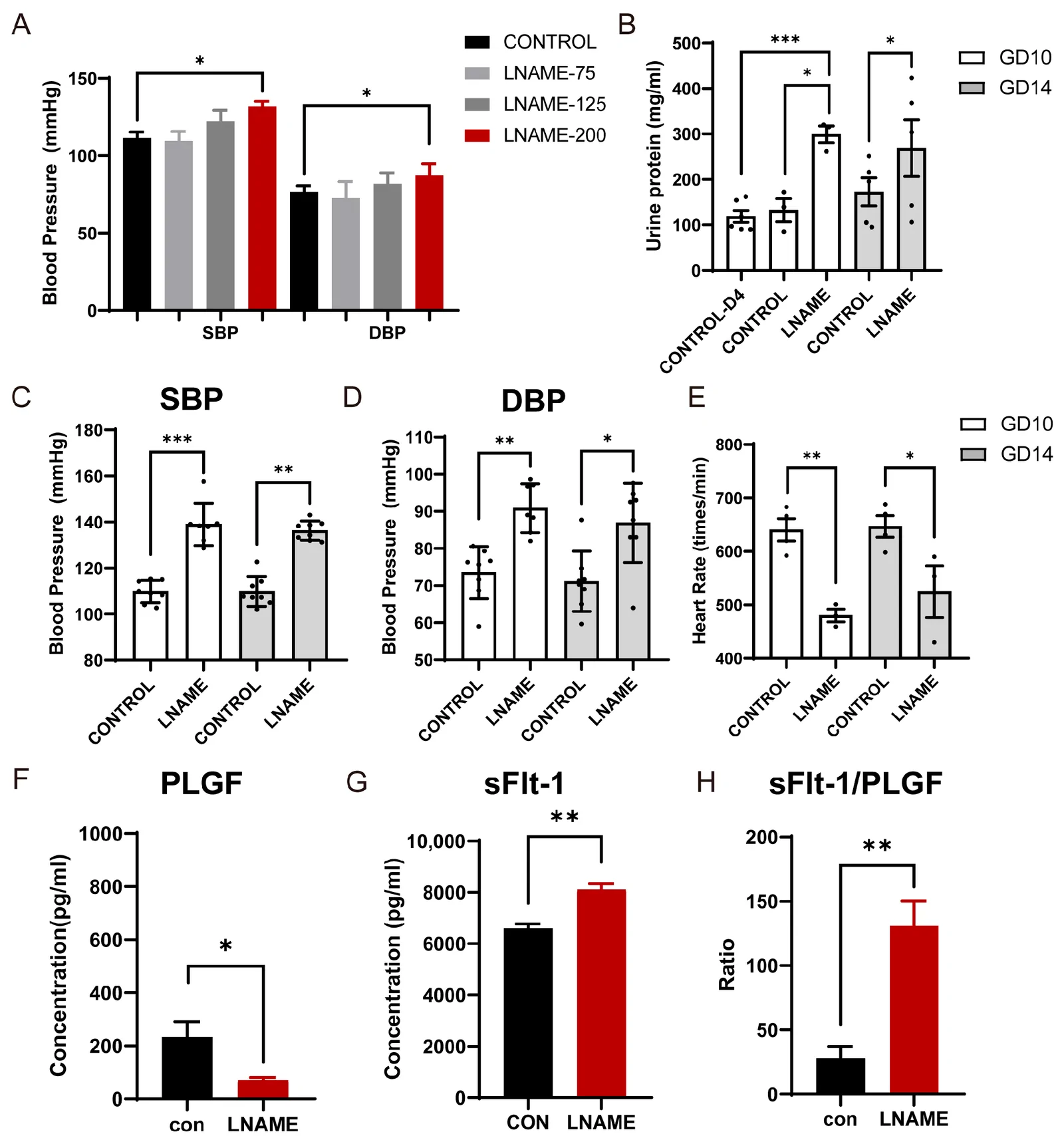
Blood pressure, urine protein and serum factors of PE mice. (**A**) Blood pressure of normal and L-NAME-treated pregnancy mice (N = 6 in each group). LNAME-75: treated with 75 mg·kg^−1^·d^−1^ L-NAME; LNAME-125: treated with 125 mg·kg^−1^·d^−1^ L-NAME; LNAME-200: treated with 200 mg·kg^−1^·d^−1^ L-NAME. (**B**) Urine protein of normal and PE mice on GD4 (N = 6), GD10 (Control: N = 3, L-NAME: N = 3) and GD14 (Control: N = 5, L-NAME: N = 5). (**C**,**D**) SBP and DBP of normal and PE mice on GD10 and GD14 (Control: N = 8, L-NAME: N = 8). (**E**) Heart rate of normal and PE mice on GD10 and GD14 (Control: N = 3, L-NAME: N = 3) (**F**–**H**) Concentration of PLGF, sFlt-1 and their ratio in normal and PE mice serum (N = 6 in each group). * *p* < 0.05, ** *p* < 0.01, *** *p* < 0.001, *t*-test.

The PE mouse model was established as previously described [28]. Three concentrations of L-NAME (75, 125, and 200 mg·kg^−1^·d^−1^) were separately injected into pregnant mice. Blood pressure measurements showed that only 200 mg·kg^−1^·d^−1^ L-NAME-induced hypertension (Figure 1A). Systolic and diastolic blood pressure of PE mice significantly increased on gestational day (GD) 10 and GD14. Additionally, their urine protein content was significantly higher than that of normal pregnant mice (Figure 1B–D). The heart rate of PE mice significantly decreased on GD10 and GD14 (Figure 1E). Placental growth factor (PLGF) levels were significantly lower, whereas soluble FMS-like tyrosine kinase 1 (sFlt-1) and PLGF/sFlt-1 levels were significantly higher in serum of PE mice than normal pregnant mice. These findings were consistent with the clinical symptoms observed in patients with PE (Figure 1F–H).

**Figure 2:**
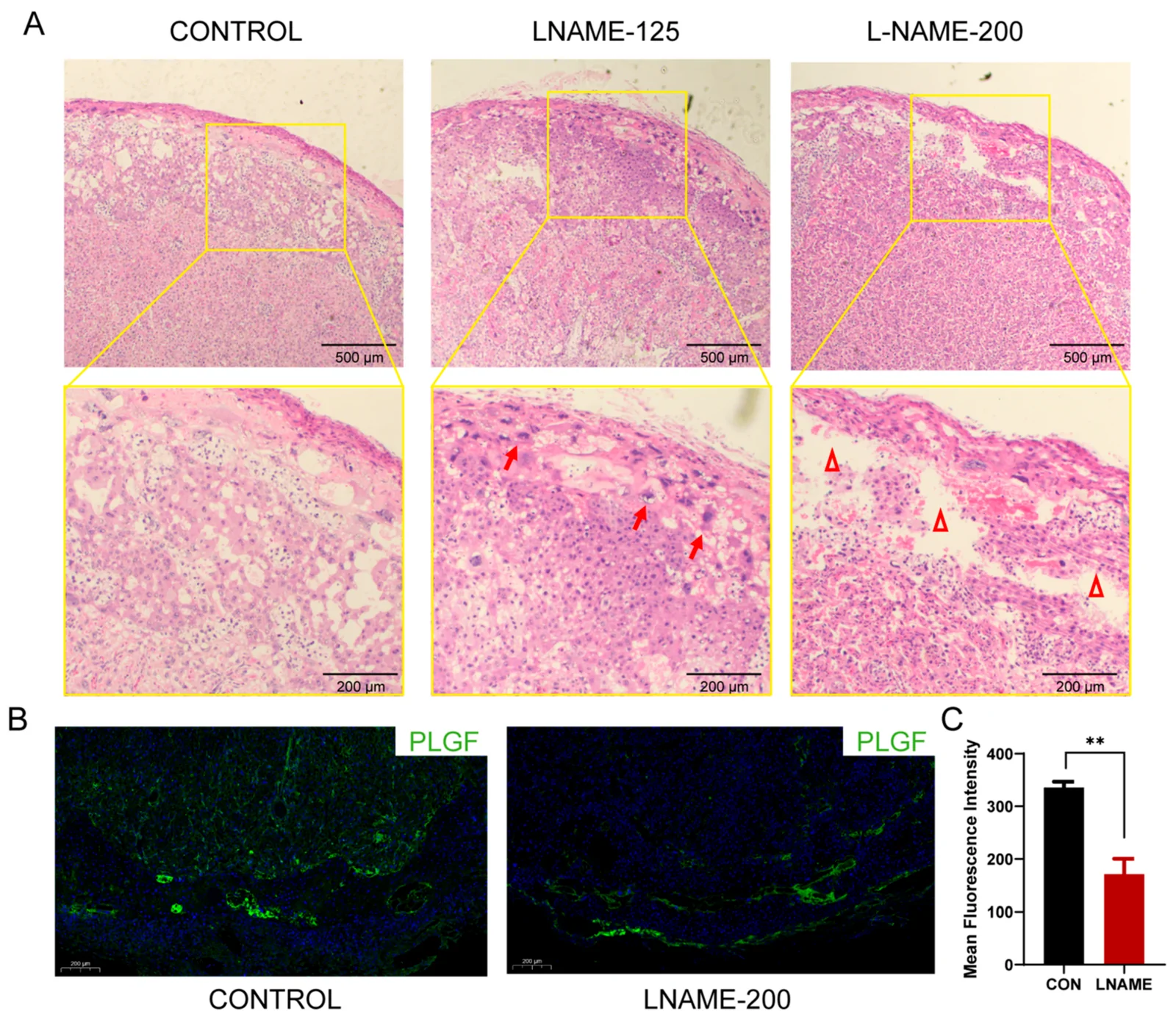
HE and IF staining of PE mice placenta. (**A**) HE staining of normal pregnant and PE mice placentae. The function zone of placenta is marked by yellow squareness. The red arrows represent cell death and the red triangles represent tissue injury. LNAME-125: treated with 125 mg·kg^−1^·d^−1^ L-NAME; LNAME-200: treated with 200 mg·kg^−1^·d^−1^ L-NAME. (**B**) IF staining of PLGF on normal pregnant and PE mice placentae. Green represents PLGF; blue represents nuclei stained by DAPI. Scale bar: 200 μm. (**C**) Statistical analysis of mean fluorescence intensity of PLGF (N = 3 in each group). ** *p* < 0.01, *t*-test.

Hematoxylin and eosin (HE) staining revealed cell death and tissue injury in the functional zone of PE placenta (Figure 2A). Immunofluorescence (IF) staining indicated that placental PLGF expression significantly decreased in PE mice compared to normal pregnant mice (Figure 2B,C), suggesting that abnormal secretion of PLGF in PE mouse serum was owing to placental dysfunction.

### 2.2. Transcriptome Sequencing Reveals the Potential Mechanism of Placental Dysfunction in PE Mice

**Figure 3:**
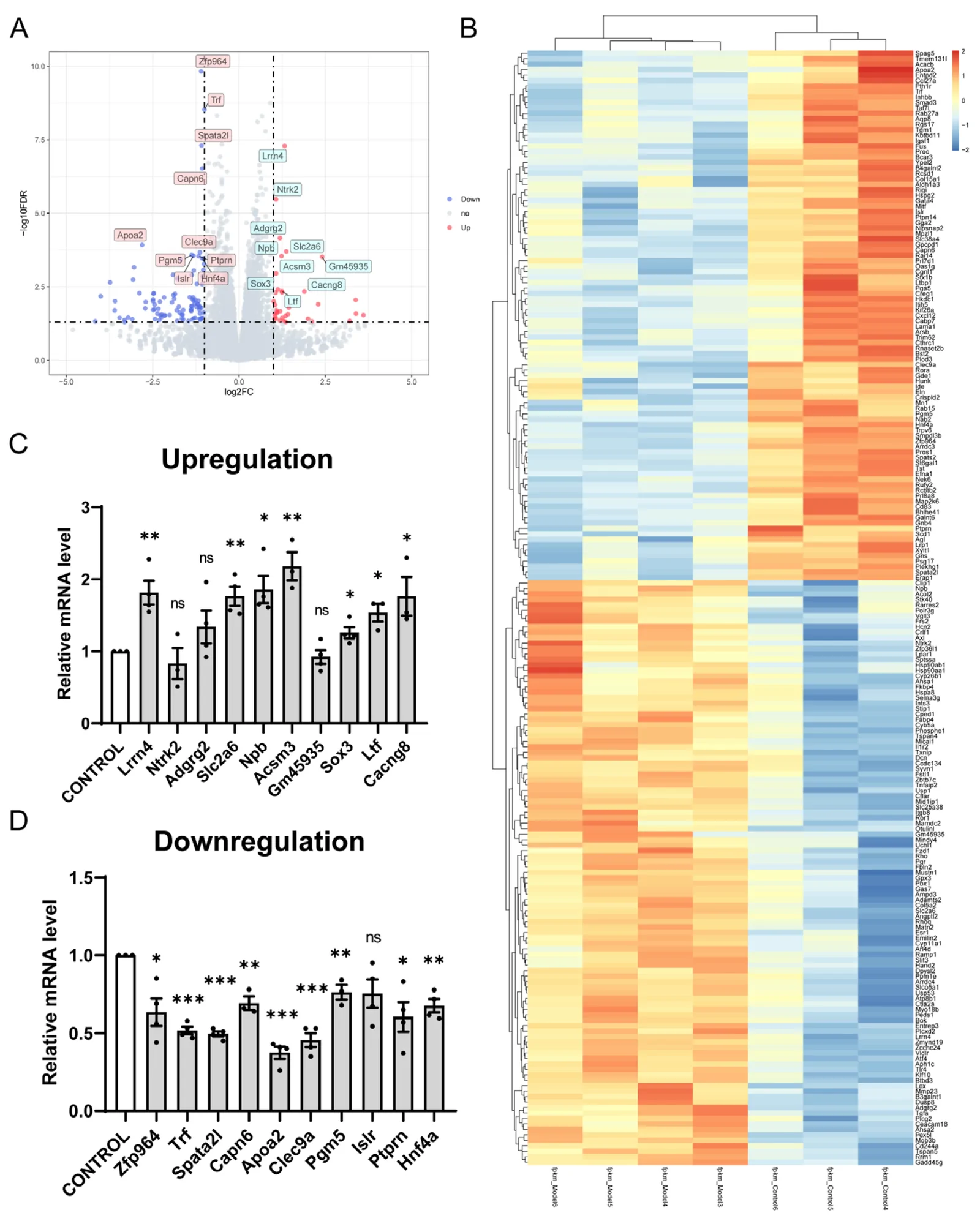
DEGs analysis of transcriptome sequencing on mice placentae. (**A**) Volcano plot of upregulated and downregulated DEGs. Dashed line: |log2FC| = 1; FDR = 0.05. (**B**) Heatmap of DEGs in control and model groups. (**C**) The expression of the top ten upregulated DEGs in normal pregnant and PE mice (Control: N = 3, L-NAME: N = 4). (**D**) The expression of the top ten downregulated DEGs in normal pregnant and PE mice (Control: N = 3, L-NAME: N = 4). * *p* < 0.05, ** *p* < 0.01, *** *p* < 0.001; ^ns^ *p* > 0.05, *t*-test.

To determine the mechanisms underlying placental dysfunction in PE mice, transcriptome sequencing was performed on normal pregnant and PE mouse placentae. A total of 122 differentially expressed genes (DEGs) were noticed, and the upregulated and downregulated DEGs are shown separately in a volcano plot (Figure 3A). The heatmap of DEGs displayed a significant difference between groups and small heterogeneity within groups (Figure 3B). The top ten significantly upregulated genes were *Lrrn4*, *Ntrk2*, *Adgrg2*, *Slc2a6*, *Npb*, *Acsm3*, *Gm45935*, *Sox3*, *Ltf*, and *Cacng8*, and the top ten downregulated genes were *Zfp964*, *Trf*, *Spata2l*, *Capn6*, *Apoa2*, *Clec9a*, *Pgm5*, *Islr*, *Ptprn*, and *Hnf4a.* Real-time quantitative polymerase chain reaction analysis showed that the expression of these genes in mouse placenta was consistent with the sequencing results (Figure 3C,D), confirming the authenticity of the transcriptome sequencing results.

**Figure 4:**
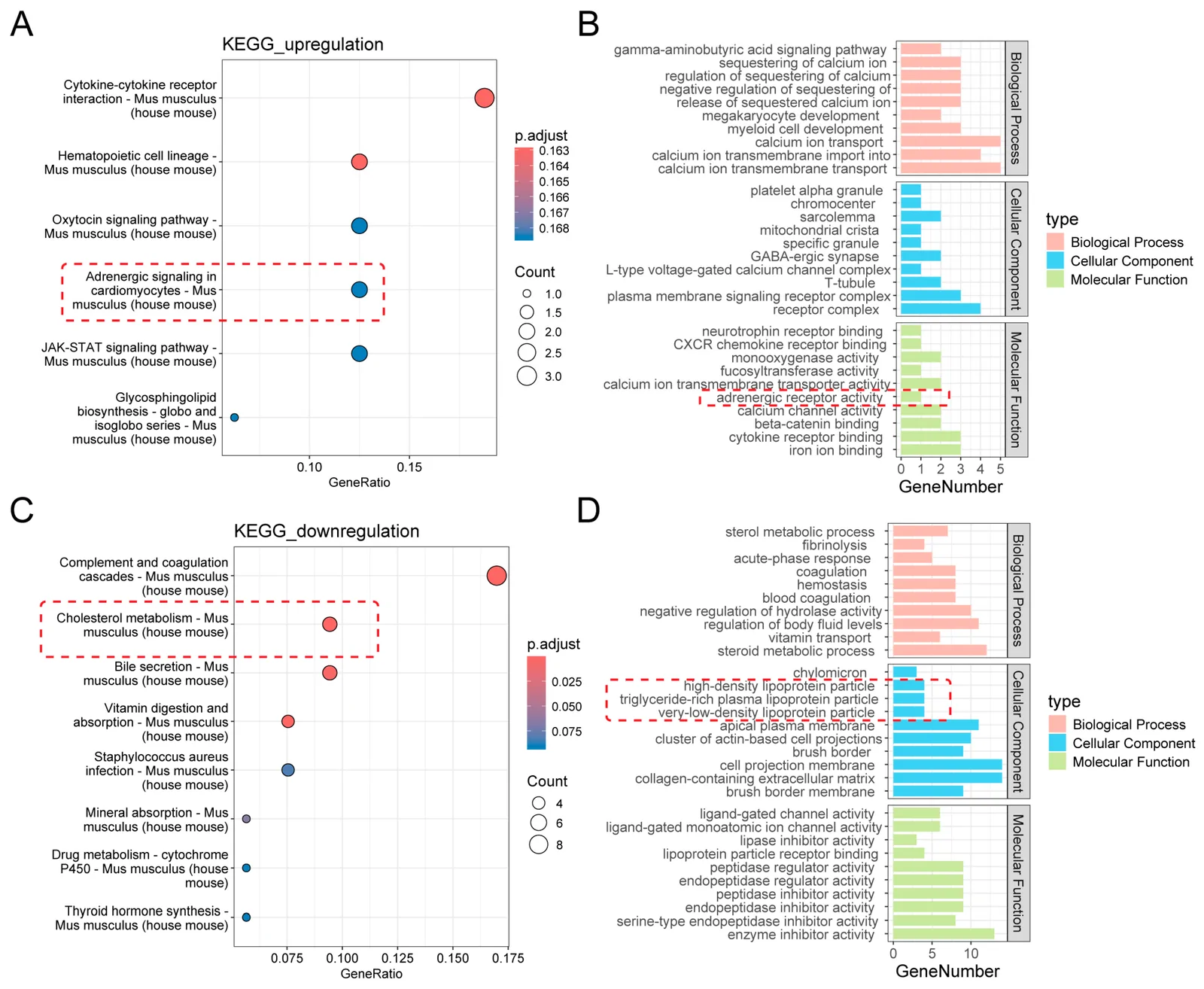
KEGG pathway and GO functional enrichment analysis of transcriptome sequencing on mice placentae. (**A**) Dot plot for KEGG enrichment of upregulated DEGs. (**B**) Bar chart for GO enrichment of upregulated DEGs. (**C**) Dot plot for KEGG enrichment of downregulated DEGs. (**D**) Bar chart for GO enrichment of downregulated DEGs. The red dashed boxes highlighted the key pathways and functional terms associated with PE placental dysfunction.

Kyoto Encyclopedia of Genes and Genomes (KEGG) pathway and Gene Ontology (GO) functional enrichment analyses were performed on upregulated and downregulated DEGs, respectively. The upregulated genes were significantly enriched in adrenergic signaling in the cardiomyocyte pathway (*p* < 0.05) and influenced molecular function of adrenergic receptor activity (Figure 4A,B). The downregulated genes were significantly enriched in the cholesterol metabolism pathway (*p* < 0.001) and influenced several lipoprotein particles (Figure 4C,D). These findings suggested that placental dysfunction in PE was associated with adrenergic receptor and metabolic disorders.

### 2.3. Integrated Transcriptomic and Network Pharmacology Analyses Prioritize ADRA1A as a Candidate TGP-Associated Target

**Figure 5:**
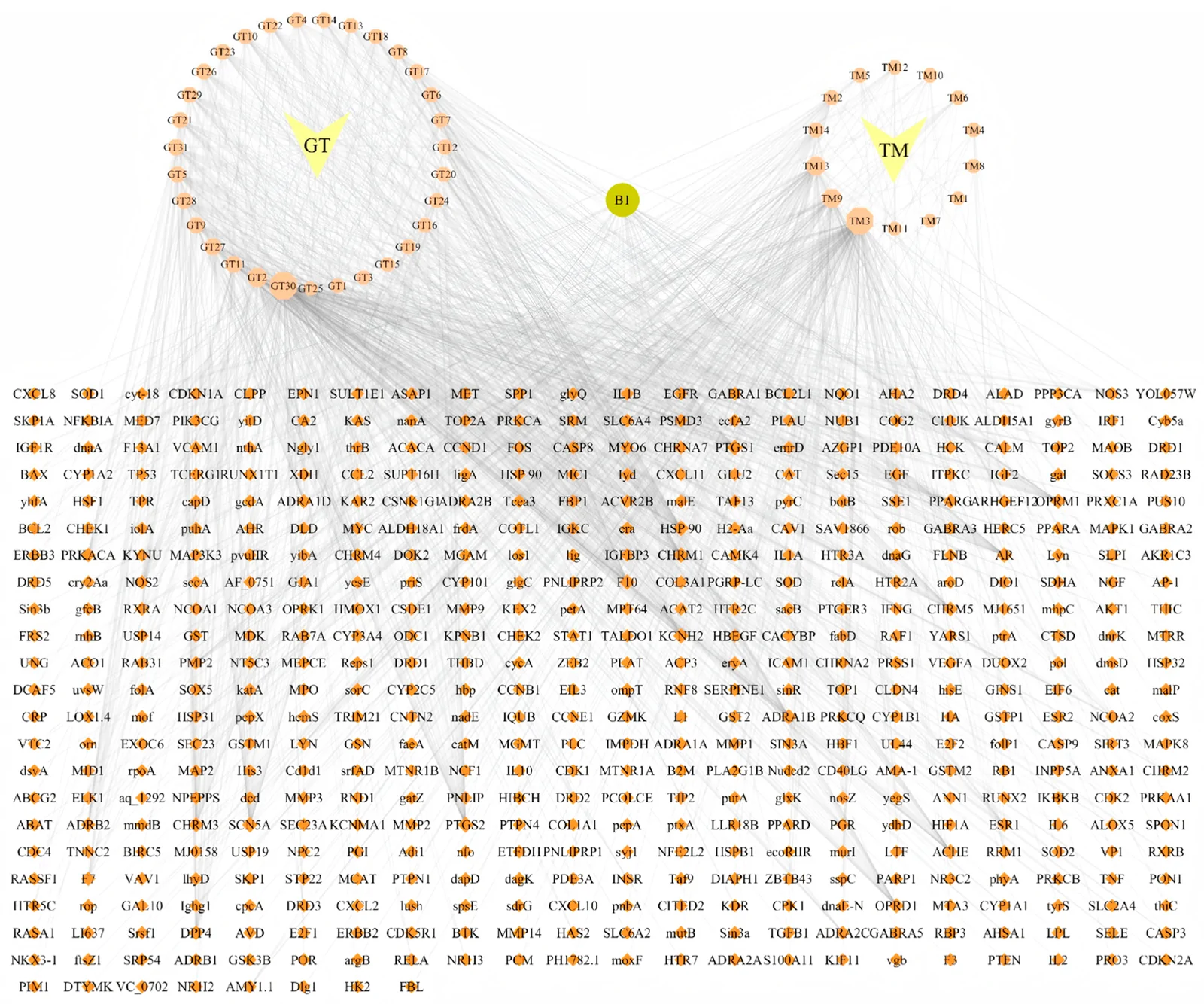
Compounds–target network diagram of TGP.

To discover the underlying mechanism of PE treatment by TGP and identify the potential bioactive compounds, network pharmacology analysis was conducted in combination with transcriptome sequencing results. The compound–target network diagram was extracted from an online database (Methods 4.5) and integrated by CytoScape (Figure 5). The diagram displayed 32 compounds from Gouteng, 15 compounds from Tianma, and 487 targets. GT1–GT31 were special compounds from Gouteng, TM1–TM14 were special compounds from Tianma, and B1 was their common compound (the name of each compound corresponding to the number is shown in Tables S1–S4).

**Figure 6:**
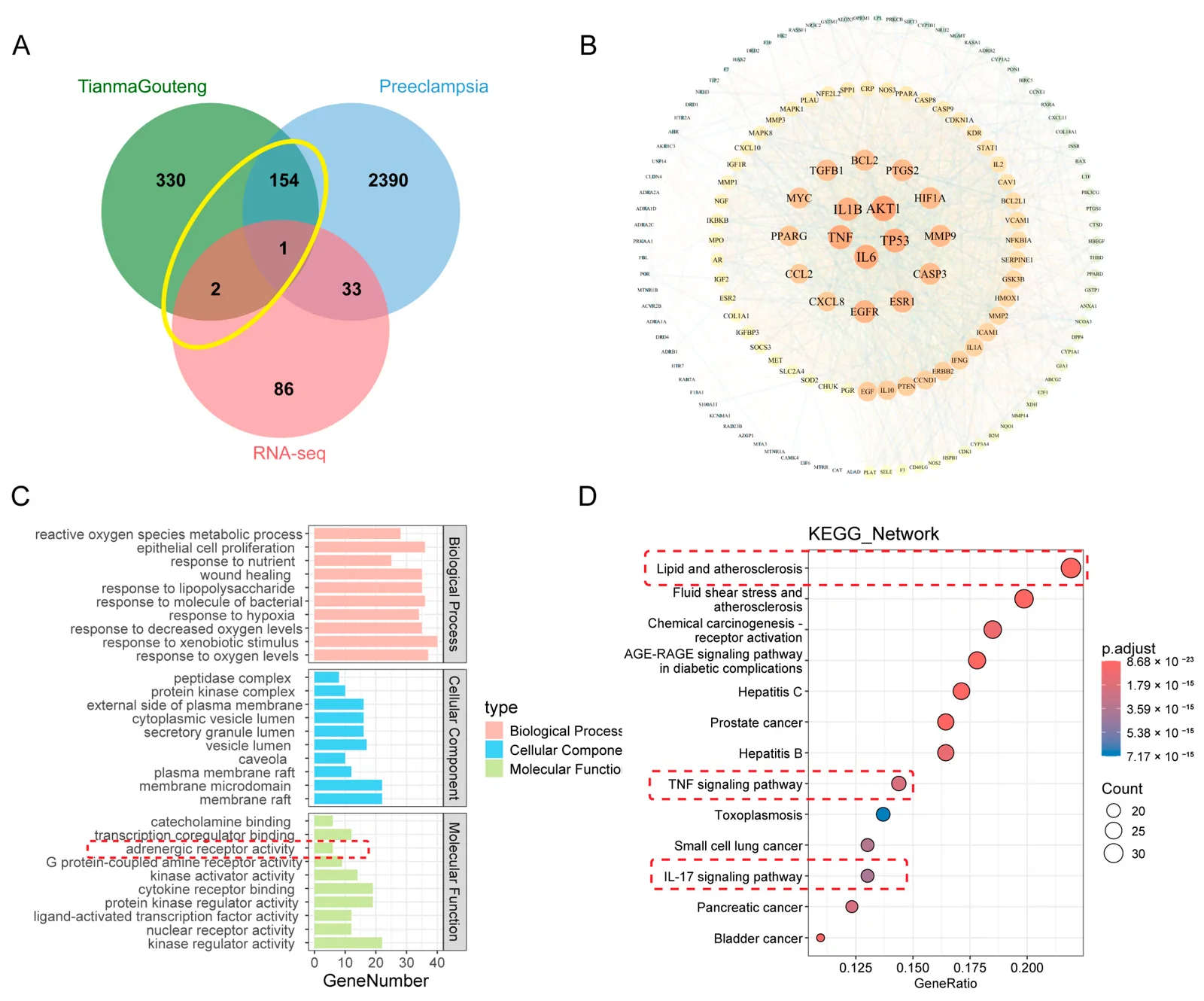
Network pharmacology analysis on TGP in the treatment of PE. (**A**) Venn diagram for overlapping targets of “drug–disease–sequencing.” (**B**) PPI network diagram of overlapping targets of “drug–disease” and “drug–sequencing.” (**C**) GO enrichment of overlapping targets. (**D**) KEGG enrichment of overlapping targets. The red box highlighted the specific enriched terms and pathways of overlapping targets.

To decipher the mechanism underlying the effect of TGP on PE placenta, Venn analysis was conducted on the targets of Tianma–Gouteng compounds (“Drug”), transcriptome sequencing (“Sequencing”), and PE disease database (“Disease”). The ternary Venn diagram showed that transcriptome sequencing contained several new targets compared to those of the existing disease database; however, its overlapping targets with those of Tianma–Gouteng were limited (Figure 6A). Since the targets of both transcriptome sequencing and PE disease database represented disease, the intersections of “Drug–Sequencing” and “Drug–Disease” were further analyzed, as shown in the yellow circle (Figure 6A). Protein–protein interaction (PPI) analysis of the overlapping targets showed that the key nodes of the network were inflammation-related factors, including interleukin 1B, tumor necrosis factor, interleukin 6, C–C motif chemokine ligand 2, and CXC motif chemokine ligand 8, and apoptosis-related factors, including caspase 3, B-cell lymphoma 2, and tumor protein 53 (Figure 6B). GO analysis showed that overlapping targets influenced adrenergic receptor activity (Figure 6C). KEGG analysis showed that the overlapping targets were significantly enriched in lipid and atherosclerosis and inflammation-related pathways, such as the TNF- and IL-17 signaling pathways (Figure 6D).

**Figure 7:**
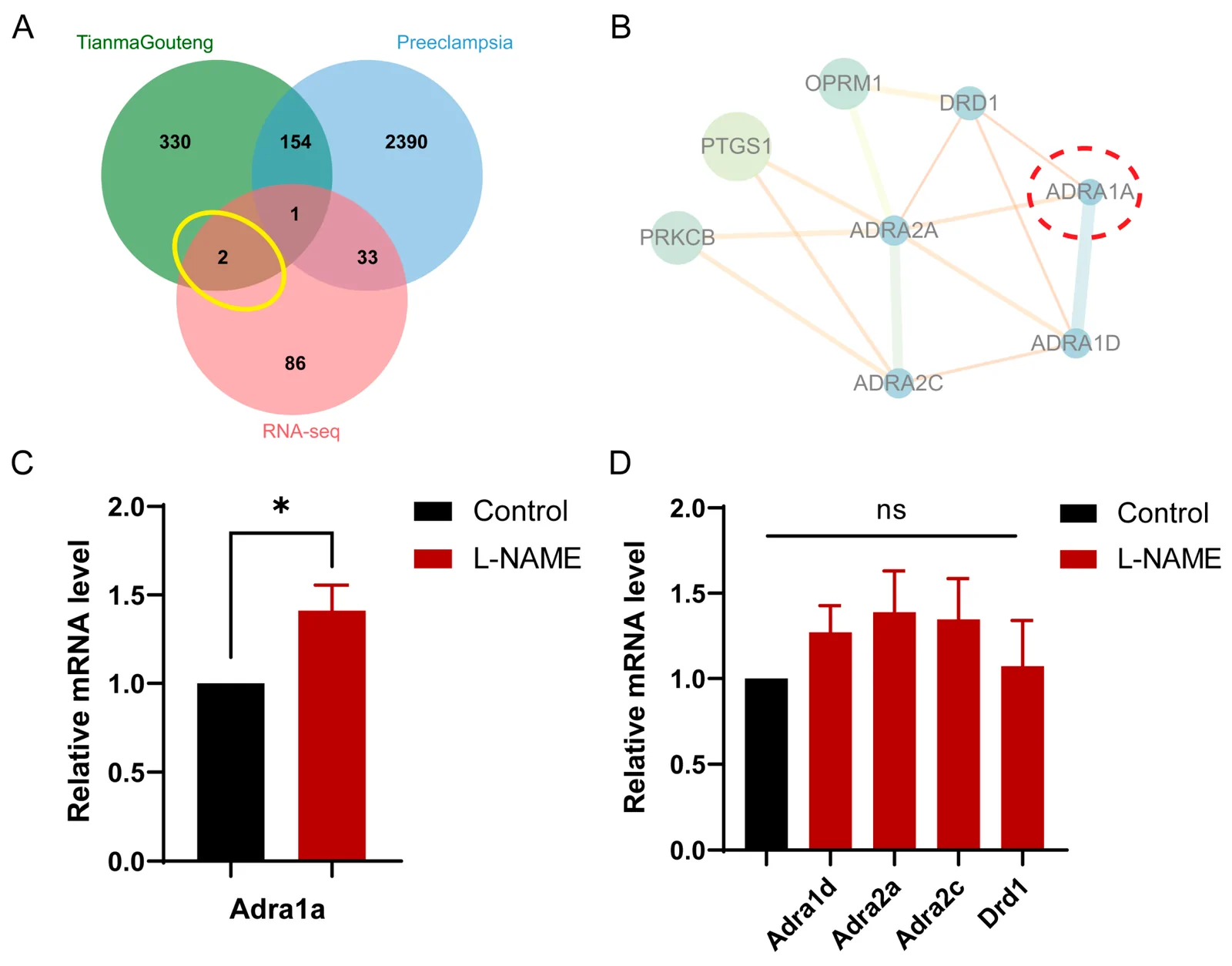
Combined transcriptome sequencing and network pharmacology analysis on TGP in the treatment of PE. (**A**) Venn diagram for overlapping targets of “drug–disease–sequencing.” (**B**) PPI network diagram of ADRA1A and its related targets. (**C**) Gene expression of *Adra1a* in normal pregnant and PE mice placentae (N = 3 in each group). (**D**) Gene expression of *Adra1d*, *Adra2a*, *Adra2c* and *Drd1* in normal pregnant and PE mice placentae (N = 3 in each group). * *p* < 0.05, ^ns^ *p* > 0.05, *t*-test.

The drug–sequencing intersection yielded two candidates as shown in the yellow circle, ADRA1A and monoamine oxidase B (MAOB). (Figure 7A). ADRA1A was advanced for focused validation because adrenergic receptor activity emerged from the placental transcriptomic enrichment analysis, ADRA1A was represented among the predicted TGP targets, and *Adra1a* expression significantly increased in PE mice placenta (Figure 7C). PPI of ADRA1A and its related targets were extracted. The network diagram showed that ADRA1A (as shown in red dashed box) interacted with various adrenergic receptor family proteins (Figure 7B). Expression of *Adra1d*, *Adra2a*, *Adra2c*, and *Drd1* also increased, but these differences were not statistically significant (Figure 7D). This was an evidence-integration step rather than a formal quantitative ranking; no composite score or PPI-centrality threshold was used to designate a primary therapeutic target. Accordingly, ADRA1A is described here as a prioritized candidate target.

### 2.4. Candidate TGP Are Associated with Reduced ADRA1A Expression in Overexpression Models

**Figure 8:**
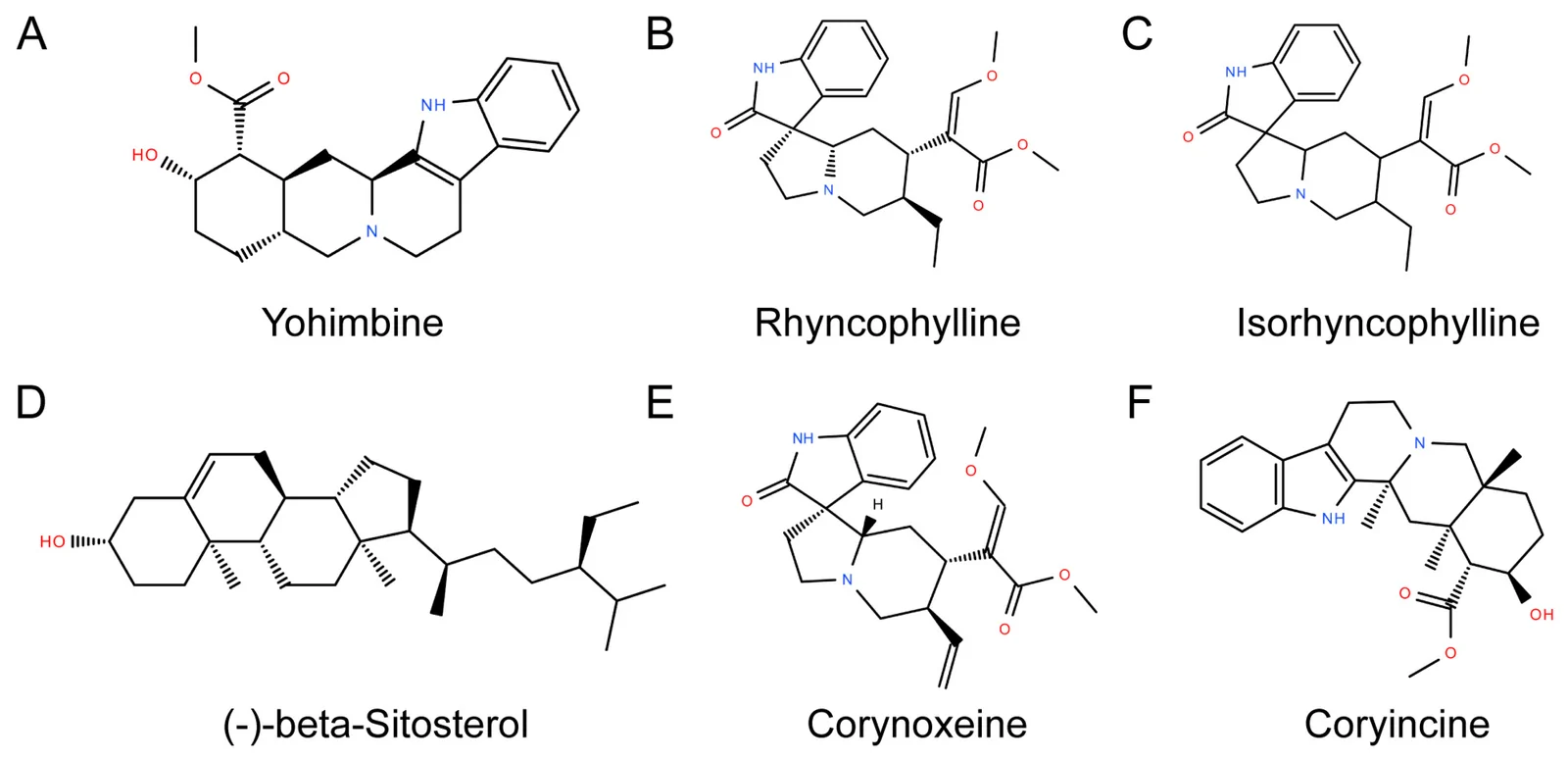
Compounds of TGP that targeted ADRA1A. (**A**) Structural formula of yohimbine. (**B**) Structural formula of rhynchophylline. (**C**) Structural formula of SRH. (**D**) Structural formula of beta-sitosterol. (**E**) Structural formula of corynoxeine. (**F**) Structural formula of coryincine.

The “compounds–target” network of TGP was used to identify six database-derived compounds predicted to interact with ADRA1A (Figure 8). Among these, five were reported for *U. rhynchophylla*—yohimbine, rhynchophylline, isorhynchophylline (SRH), corynoxeine, and coryincine—and beta-sitosterol was reported for both *G. elata* and *U. rhynchophylla*. Because coryincine was reported at relatively low abundance in *U. rhynchophylla*, the remaining five compounds were advanced for experimental evaluation. The validation experiments used purchased purified compounds rather than a standardized TGP extract.

**Figure 9:**
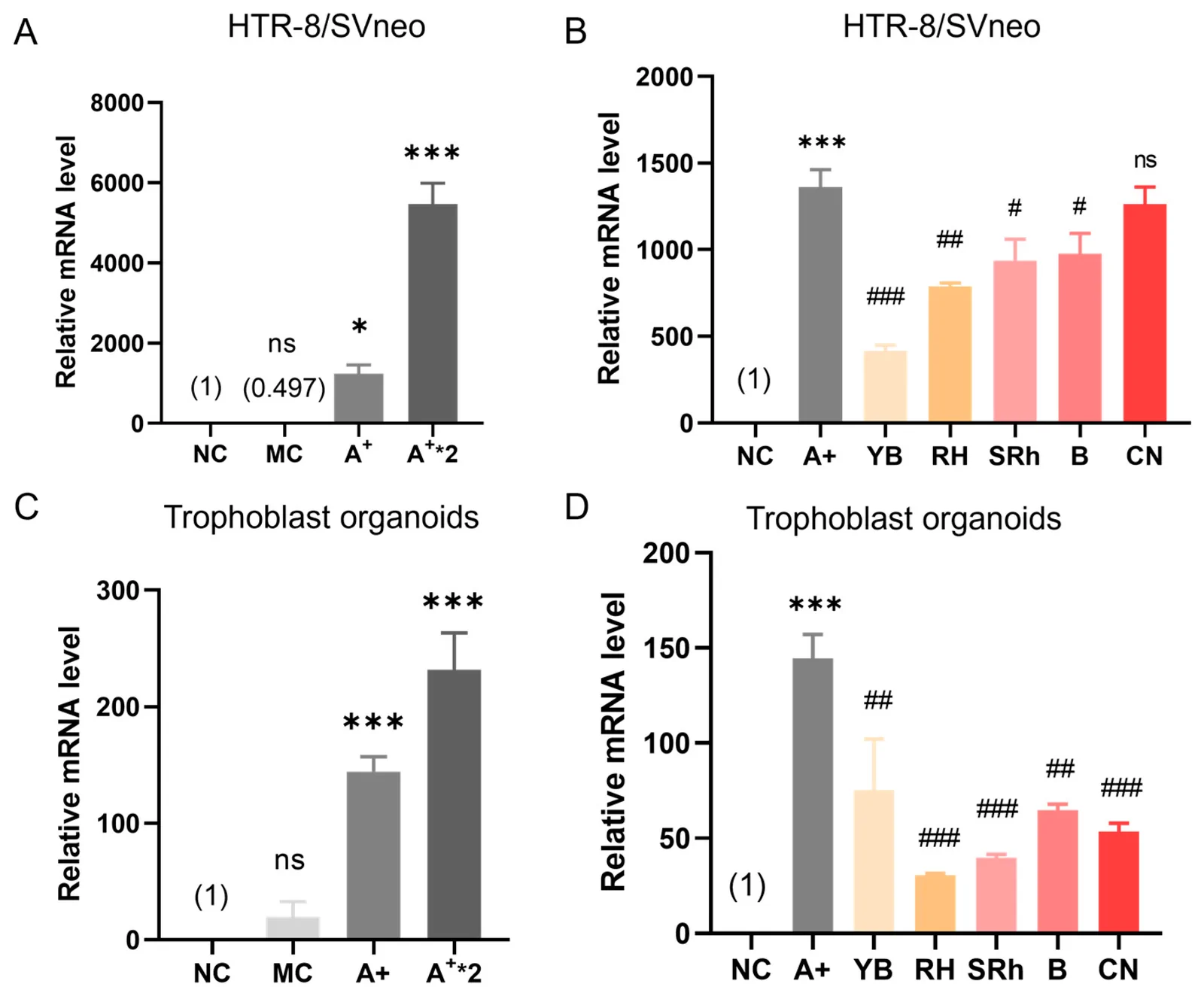
Experimental validation of biological activity of compounds from Tianma–Gouteng. (**A**) Expression of *ADRA1A* in ADRA1A-overexpressed HTR-8/SVneo cells (N = 3 in each group). (**B**) Expression of *ADRA1A* in ADRA1A-overexpressed HTR-8/SVneo cells treated with bioactive compounds (N = 3 in each group). (**C**) Expression of *ADRA1A* in ADRA1A-overexpressed trophoblast organoids (N = 3 in each group). (**D**) Expression of *ADRA1A* in ADRA1A-overexpressed trophoblast organoids treated with bioactive compounds (N = 3 in each group). NC: negative control; MC: mock control; A^+^: ADRA1A overexpression; A^+^*2: double plasmid treated ADRA1A overexpression. YB: yohimbine; RH: rhynchophylline; SRH: isorhyncophylline; B: beta-sitosterol; CN: corynoxeine. The mRNA levels are expressed as fold changes relative to the NC group. The value for the NC group is indicated as (1) in the figure. * *p* < 0.05, *** *p* < 0.001 vs. the control group; ^#^ *p* < 0.05, ^##^ *p* < 0.01, ^###^ *p* < 0.001 vs. the model group; ^ns^ *p* > 0.05, One-way ANOVA.

*ADRA1A*-overexpressing HTR-8/SVneo cells and trophoblast organoids were used to evaluate whether the selected compounds were associated with changes in ADRA1A expression. Low and high (2×) dosages of the plasmid-treated overexpression model were generated. Both dosages significantly increased *ADRA1A* expression in HTR-8/SVneo cells (Figure 9A, A^+^: *p* = 0.0345, A^+^*2: *p* < 0.0001; R^2^ = 0.9697). Therefore, subsequent validation experiments were conducted using the low-dose plasmid-treated overexpression model. The overexpression of *ADRA1A* in HTR-8/SVneo cells was significantly rescued after treatment with yohimbine, rhynchophylline, SRH, and beta-sitosterol (Figure 9B, SRH: *p* = 0.0035; R^2^ = 0.9357). However, the *ADRA1A* expression after corynoxeine treatment was not significantly decreased.

Trophoblast organoid experiments showed a similar expression pattern. Trophoblast organoids were constructed as previously described [32]. Low and high (2×) dosages of the plasmid-treated overexpression model were established, and both dosages significantly increased *ADRA1A* expression in trophoblast organoids (Figure 9C, A^+^: *p* = 0.0004, A^+^*2: *p* < 0.0001; R^2^ = 0.9559). Moreover, all five compounds rescued *ADRA1A* expression in trophoblast organoids (Figure 9D, SRH: *p* = 0.0004; R^2^ = 0.9343), with larger changes observed for rhynchophylline and SRH in this assay. These results were used to select compounds for subsequent binding and injury-model studies.

### 2.5. SRH Directly Binds to ADRA1A and Is Associated with Partial Restoration of Typical Markers in an L-NAME-Induced Trophoblast Organoid Injury Model

Since the five compounds produced different ADRA1A-expression changes, molecular docking was used as an additional screening step. The predicted binding energies from high to low were beta-sitosterol, SRH, yohimbine, corynoxeine, and rhynchophylline (Table 1). Considering the expression results together with the docking prediction, SRH was selected as a lead compound for following validation experiments.

**Figure 10:**
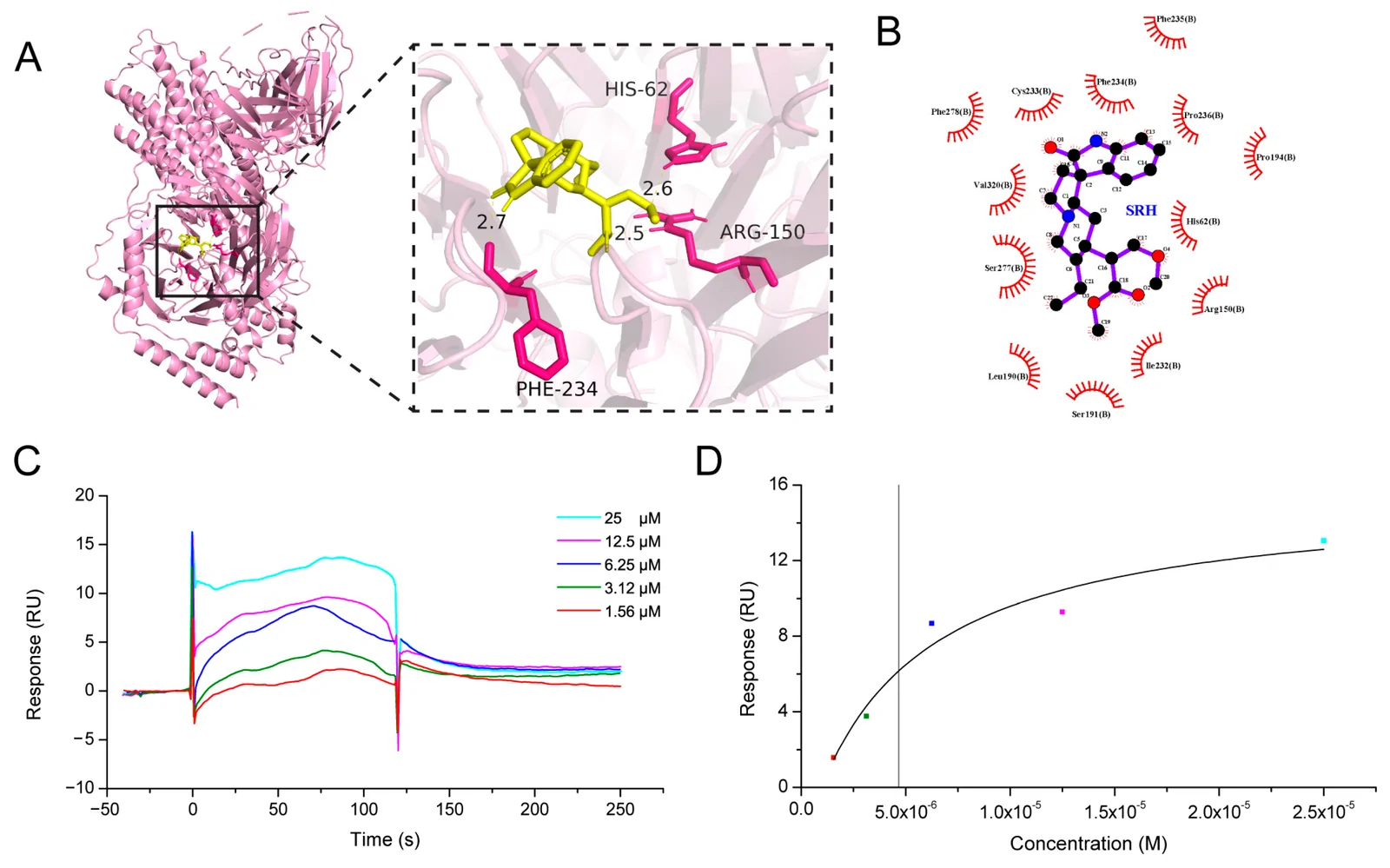
Molecular docking and experimental validation. (**A**) Overall structural diagram of SRH and ADRA1A and 3D schematic diagram of hydrogen bonding. (**B**) 2D schematic diagram of hydrogen bonds. (**C**,**D**) SPR results of SRH and ADRA1A.

Molecular docking predicted hydrogen-bond interactions between SRH and HIS-62, ARG-150, and PHE-234 residues of ADRA1A (2.6, 2.5, and 2.7 Å, respectively; Figure 10A,B). SPR yielded an equilibrium affinity estimate of KD = 4.67 μM for SRH binding to immobilized ADRA1A (Figure 10C,D). These results support measurable physical binding under the assay conditions.

**Figure 11:**
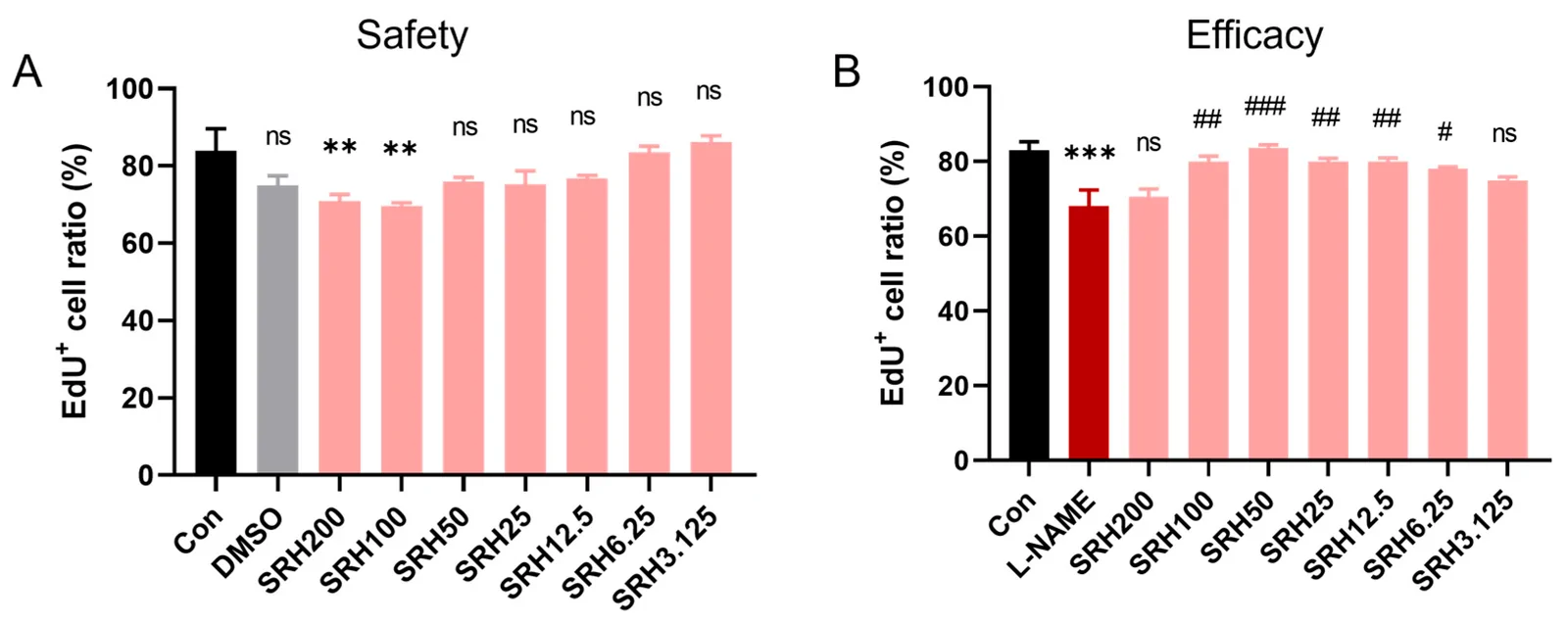
Safety and efficacy evaluation of SRH in HTR-8/SVneo cells. (**A**) EdU staining of HTR-8/SVneo cells after dose-gradient SRH treatment (N = 3 in each group). (**B**) EdU staining on L-NAME-induced injury HTR-8/SVneo cells after dose-gradient SRH treatment (N = 3 in each group). ** *p* < 0.01, *** *p* < 0.001 vs. the control group; ^#^ *p* < 0.05, ^##^ *p* < 0.01, ^###^ *p* < 0.001 vs. the model group; ^ns^ *p* > 0.05, one-way ANOVA.

Dose-dependent safety and efficacy evaluation of SRH was conducted on HTR-8/SVneo cells. The 5-ethynyl-2′-deoxyuridine (EdU) staining results of safety evaluation showed that the EdU^+^ cell proportion was not significantly decreased at an SRH concentration of less than 100 μM (Figure 11A, R^2^ = 0.6857), indicating that the proliferation ability of SRH remained unaltered. EdU staining further showed that SRH significantly restored the EdU^+^ cell proportion of L-NAME-induced injury cells at a concentration between 6.25 μM and 100 μM (Figure 11B, R^2^ = 0.7817), suggesting the recovery of cell proliferation. At an SRH concentration of 25 μM, the EdU^+^ cell proportion was the closest to that of the control group.

**Figure 12:**
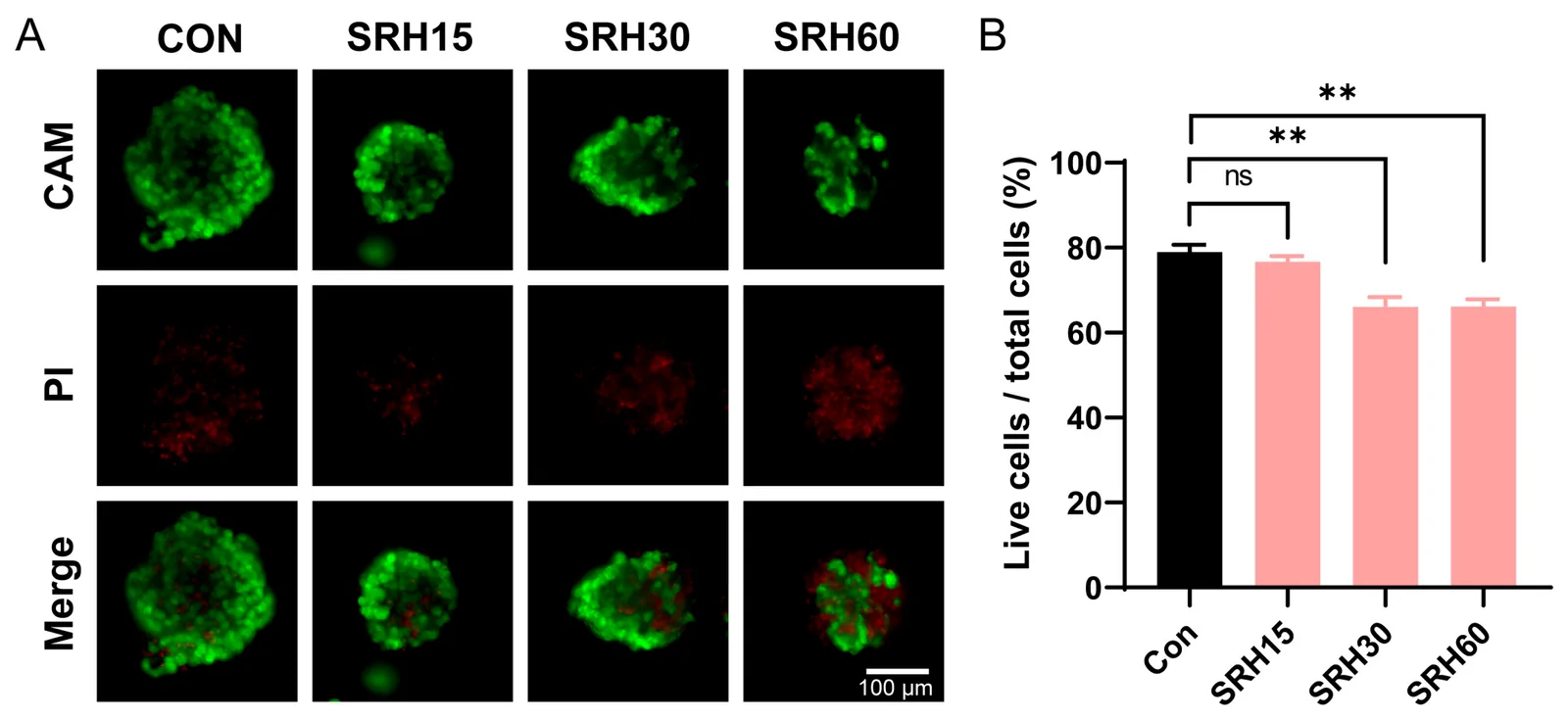
Safety evaluation of SRH on trophoblast organoids. (**A**) Live–dead cell staining on trophoblast organoid after SRH treatment. Green represents the live cells stained by calcein AM; red represents the dead cells stained by PI. CON: control; SRH15: treated with 15 μM SRH; SRH30: treated with 30 μM SRH; SRH60: treated with 30 μM SRH. (**B**) Statistical analysis of live–dead cell staining (N = 3 in each group). ** *p* < 0.01; ^ns^ *p* > 0.05, *t*-test.

Safety evaluation of SRH was conducted using trophoblast organoids. Live–dead cell staining indicated that the proportion of live cells was not significantly different between control and 15 μM SRH-treated groups (Figure 12A,B, SRH15: *p* = 0.7603, SRH30: *p* = 0.0001, SRH60: *p* = 0.0004; R^2^ = 0.3142). However, the proportion of live cells significantly decreased after 30 or 60 μM SRH treatment. These results suggested that high concentrations of SRH aggravated placental injury.

**Figure 13:**
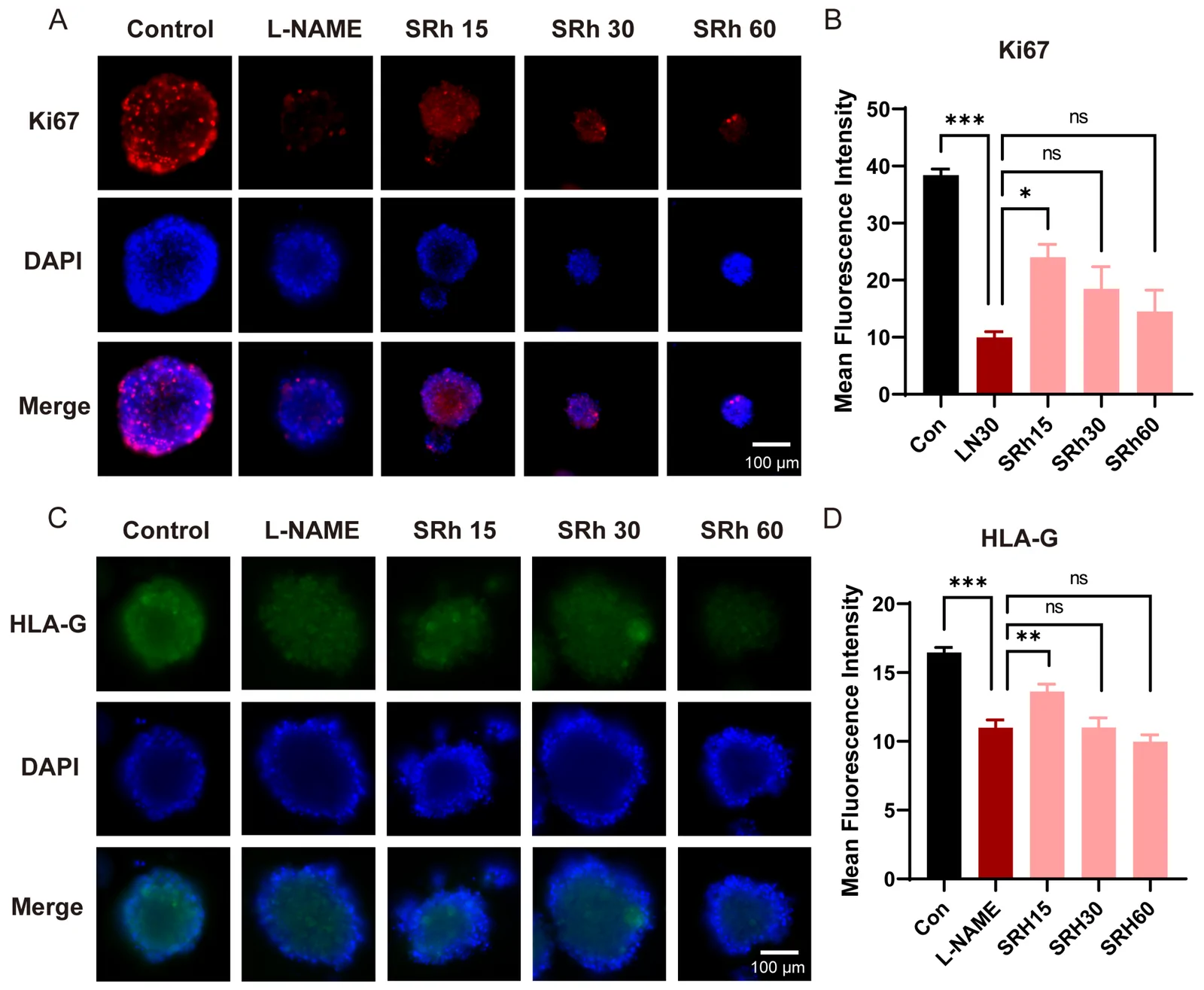
Protective effects of SRH on L-NAME-induced trophoblast injury model. (**A**) IF staining of Ki67 in L-NAME-induced trophoblast organoid injury model treated by SRH. Red represents Ki67; blue represents nuclei stained by DAPI. (**B**) Statistical analysis of IF staining (N = 3 in each group). (**C**) IF staining of HLA-G in L-NAME-induced trophoblast organoid injury model treated by SRH. Green represents HLA-G; blue represents nuclei stained by DAPI. (**D**) Statistical analysis of IF staining (N = 3 in each group). * *p* < 0.05, ** *p* < 0.01, *** *p* < 0.001; ^ns^ *p* > 0.05, one-way ANOVA.

An L-NAME-induced trophoblast organoid injury model was established to evaluate typical trophoblast phenotypes. The levels of the proliferation-related indicator Ki67 and differentiation-related indicator human leukocyte antigen-G (HLA-G) were analyzed to evaluate the protective effects of SRH. IF staining results indicated that Ki67 and HLA-G expression significantly decreased in L-NAME-induced trophoblast organoids, which was significantly rescued by 15 μM SRH treatment (Figure 13A–D, SRH15: *p* = 0.0394, R^2^ = 0.7942; SRH15: *p* = 0.0027, R^2^ = 0.4795). These results suggested SRH was associated with partial restoration of the typical proliferation and differentiation markers in the injury model.

## 3. Discussion

### 3.1. Summary of Findings

PE is a common pregnancy-related disorder. It has become an urgent public safety concern owing to its high incidence and various adverse pregnancy outcomes. As a classic anti-hypertensive prescription in TCM, the efficacy of TGP has been validated in clinical trials and evidence-based medicine [33,34,35], including its attenuation of PE-related pathological features such as hypertension, proteinuria, and placental injury. In this study, we identified potential bioactive compounds in TGP for treating PE, which were mostly natural alkaloids from *U. rhynchophylla*. These natural compounds potentially targeted ADRA1A, a candidate target that modulates PE progression. Among these compounds, SRH directly bound to ADRA1A and demonstrated a protective effect against L-NAME-induced trophoblast cell and organoid injury.

### 3.2. Bioactive Compounds of TGP

To date, most pharmacological investigations have focused on the active constituents of *G. elata* or *U. rhynchophylla* individually. In contrast, the bioactive components of TGP—and their potential therapeutic effects in PE—have received little attention. While clinical trials and evidence-based medicine have confirmed the therapeutic efficacy of TGP-containing formulations in treating PE, additional research is required to clarify the relevant bioactive compounds responsible and to elucidate their pharmacological mechanisms.

In this study, five bioactive compounds targeting ADRA1A were identified from *U. rhynchophylla*, among which only β-sitosterol was a component common to both *G. elata* and *U. rhynchophyll*. Based on the *Chinese Materia Medica*, *U. rhynchophylla* is known for its effects of calming wind, stopping spasms, clearing heat, and soothing the liver, and is primarily used to treat eclampsia. *G. elata* also possesses these effects; however, it is mainly used for treating acute and chronic convulsions and seizures [36,37]. Modern pharmacological studies have revealed that *U. rhynchophylla* and its main alkaloids can lower blood pressure [38,39,40], and *G. elata* and its main component, gastrodin, are frequently used in treating neurological disorders such as migraines [41,42]. Therefore, although both *G. elata* and *U. rhynchophylla* were the principal herbs in the same prescription, they had different therapeutic functions and exerted different effects on the complex clinical symptoms of PE. In the present study, the mouse model of PE used for transcriptome sequencing mainly simulated conditions, such as hypertension, proteinuria, and placental dysfunction, of PE and was unable to replicate symptoms, such as headache and convulsions; therefore, the active compounds identified were primarily from *U. rhynchophylla*, which corresponded to its therapeutic effects and pharmacological actions. Consequently, optimizing animal models of PE or using clinical samples for sequencing analysis will be crucial to validate the current findings and further explore the bioactive components of TGP relevant for the treatment of PE.

### 3.3. Target of TGP in the PE Placentae

In recent years, an expanding body of literature has elucidated the association between placental dysfunction and the pathophysiology of PE. However, the translation of such mechanism into clinical therapies or their application to refine paradigms remains limited. In the present study, ADRA1A was identified as a candidate target of TGP in PE placentae. ADRA1A is a subtype of α1-adrenergic receptors, and primarily mediates vasoconstriction and smooth muscle contraction in response to catecholamines [43]. Previous studies on adrenergic signaling mostly focused on its regulation of the cardiovascular system, including in PE [20]. Our findings align with those of previous studies showing that ADRA1A regulates human placental vessel contractility [18]. Notably, our study extends these observations by identifying specific natural compounds that modulate ADRA1A expression in trophoblasts. Future studies should use clinical samples and multi-omics sequencing to delineate the roles of ADRA1A and adrenergic pathways in the pathological progression of PE.

MAOB, was also identified as a candidate target. However, MAOB was primarily associated with neurotransmitter metabolism and had weaker mechanistic links to PE placental pathology [44]. The PPI network analysis also defined inflammation- and apoptosis-related factors contributing to TGP’s therapeutic effects. However, owing to technical limitations related to drug screening for multiple targets, other targets were not included in this study for drug screening. Future studies should investigate whether TGP compounds also modulate these targets, either directly or indirectly, and whether these pathways interact with ADRA1A signaling. A comprehensive analysis of the full PPI network using multi-omics approaches would provide a more complete picture of the mechanism underlying TGP in the treatment of PE.

### 3.4. Innovation Based on Trophoblast Organoids

Human villus-derived trophoblast organoids, in vitro models of the placenta with three-dimensional structures, feature relatively highly complex cell types and microenvironments and have been widely used in studies on the pathogenesis of obstetric diseases and pharmacology and toxicology during pregnancy [45,46]. In the present study, trophoblast organoids were successfully constructed from multiple human villi and passaged for over 20 generations (approximately 150 days), indicating a stable long-term culture. Although the methods for constructing trophoblast organoids are relatively advanced, decidual contamination continues to occur. This issue has been mainly addressed by controlling the digestion time of villous tissue and using mild digestive enzymes [7].

Several in vitro models, such as cell line and placental explant models, have been used to study placental dysfunction [47,48,49]; however, most lack complex cell types and intercellular microenvironments. Trophoblast organoids derived from early-onset PE placentae has also been reported in the study of PE [50]; however, the early-onset PE placentae in that study were derived from patients at 28–34 weeks of gestation. Therefore, the respective organoids could not capture the pathological changes of PE placentae at an early stage. The hypoxia-induced trophoblast organoid model also imitates the pathophysiology of PE [51]; however, the L-NAME-induced injury model is likely more stable than the hypoxia-induced models. The PLGF and sFlt-1 levels in the supernatant of L-NAME-induced injury trophoblast organoids detected in our previous study were consistent with those in the serum of patients with PE, indicating that this organoid injury model is representative of placental dysfunction in PE. In future research, transcriptome sequencing or single-cell transcriptomics could be conducted using the current model, and a comparative analysis with sequencing results from clinical PE samples could be performed to clarify the shortcomings of the existing models and expand the application scenarios of trophoblast organoid-injury models.

### 3.5. Limitations of the Study

The current study has several limitations. Firstly, the trophoblast organoids established lacked vascular structure, which is a crucial component of the placenta and plays a significant role in placental function [7]. As drugs targeting ADRA1A to treat hypertension primarily exert their effects by regulating the contraction of blood vessels and smooth muscles, vascular structure is vital for studying the mechanisms underlying TGP and SRH. Secondly, although SRH downregulated the expression of *ADRA1A* and directly bound to ADRA1A, whether this mechanism underlies the effects of SRH on PE needs verification. Finally, the current study was based on computational prediction and preclinical validation, and validation in living organisms remains crucial. In silico predictions are subject to potential bias and may fail to capture all relevant targets. Moreover, the extrapolation of preclinical results to clinical settings should be interpreted with caution.

To further validate the mechanism underlying TGP, future studies employing ADRA1A knockdown or knockout in trophoblast organoids combined with TGP or SRH treatment will be essential to definitively establish that the protective effects of TGP and SRH are mediated specifically through ADRA1A. Additionally, ADRA1A-knockout mouse models would enable in vivo validation of the therapeutic mechanism. Secondly, an in vivo study on SRH and other bioactive compounds from TGP could be employed to validate their efficacy. Finally, the vascularization improvement of trophoblast organoids may be achieved by introducing a coculture system with vascular cells or organoids [52] and developing bioengineered organoid-on-chip platforms [53]. Such investigations would further promote the clinical application of TGP; however, clinical research on the human placenta are warranted to enhance the translational value of these findings.

## 4. Materials and Methods

### 4.1. Materials and Reagents

SRH (CAS: 6859-01-4, purity ≥ 98%), yohimbine (CAS: 131-03-3, purity ≥ 98%), rhynchophylline (CAS: 76-66-4, purity ≥ 98%), corynoxeine (CAS: 630-94-4, purity ≥ 98%), and beta-sitosterol (CAS: 83-46-5, purity ≥ 98%) were purchased from Shanghai Winherb Medical Science Co., Ltd. (Shanghai, China). L-NAME was provided by Beyotime Biotechnology (ST1555, purity ≥ 98%). The antibodies against Ki67 (ab279693), HLA-G (ab283260) were provided by Abcam (Cambridge, UK). Alex 488-conjugated and Alex 647-conjugated secondary antibodies (A0423, A0473) were provided by Beyotime Biotechnology (Shanghai, China).

### 4.2. Animal Experiments

The female C57BL/6J mice with mating plug were obtained from Hangzhou Enlighten the Truth Laboratory Animal Technology Co., Ltd. (Hangzhou, China; certification SCXK (Zhejiang) 2022-0050). The PE mouse model was established by administering L-NAME into pregnant mice on GD8–GD14 through subcutaneous injection. Three concentrations (75, 125 and 200 mg·kg^−1^·d^−1^) of L-NAME were tested and only 200 mg·kg^−1^·d^−1^ L-NAME-induced hypertension was finally used. The blood pressure and urine protein of pregnant mice were measured on GD10 and GD14 as previously described [28].

### 4.3. Enzyme-Linked Immunosorbent Assay (ELISA)

The maternal serum was obtained from pregnant mice on GD 15. The concentrations of serum factors PLGF and sFlt-1 were measured according to the manufacturer’s protocol of a Mouse PlGF-2 Quantikine ELISA Kit (R&D, Minneapolis, MN, USA, MP200) and Mouse VEGFR1/FIt-1 Quantikine ELISA Kit (R&D, Minneapolis, MN, USA, MVR100).

### 4.4. HE Staining

The placentae samples were obtained from pregnant mice on GD 15. Fixed placenta samples were embedded in paraffin and sectioned at a thickness of 5 μm using a microtome. The sections were conducted with deparaffinization, hydration, and staining with HE regent.

### 4.5. IF Staining

Trophoblast organoids were fixed with 4% polyformaldehyde directly in 96-well culture plate with black transparent bottom and stained as previously described [54]. The stained trophoblast organoids were captured by laser confocal microscopy (Leica, Wetzlar, Germany) or an ImageXpress Pico Automated Cell Imaging System (Molecular Devices, San Jose, CA, USA). The mean fluorescence intensity of images was calculated using ImageJ 1.53.

Paraffin-embedded placenta sections were treated with deparaffinization, hydration, and staining with anti-PLGF antibody and fluorescent secondary antibody.

### 4.6. Transcriptome Sequencing

Total RNA was extracted from approximately 20 mg of placental tissue obtained from control (N = 3) and preeclampsia mice (N = 4) using a TRIzol regent kit (Invitrogen, Waltham, MA, USA, 12183555CN).

RNA integrity was assessed was assessed using an Agilent 2100 Bioanalyzer with RIN ≥ 7.0 as the acceptance threshold. Library construction and sequencing were performed by BGI Genomics (Shenzhen, China). Raw reads were filtered to remove reads containing adapters, reads with >10% ambiguous bases, and low-quality reads (Q ≤ 10) using SOAPnuke v2.0. Clean reads were aligned to the mouse reference genome (GRCm38) using HISAT2 (version 2.1.0) with default parameters. Gene-level read counts were quantified using feature Counts, and expression levels were normalized as FPKM (Fragments Per Kilobase of transcript per Million mapped reads). Differential expression analysis (|log2FoldChange| ≥ 1; FDR-adjusted *p*-value (Benjamini-Hochberg) < 0.05) between control and model groups was conducted using the DESeq2 package (version 1.46.0) in R (version 4.4.2). Heatmaps were generated with pheatmap (version 1.0.12), pathway enrichment analysis with clusterProfiler (version 4.14.4), and visualization with ggplot2 (version 4.0.1). All statistical analyses were performed under R environment.

### 4.7. Network Pharmacology Analysis

Active compounds from *U. rhynchophylla* were screened in the TCMSP database (https://www.tcmsp-e.com/tcmsp, access data: 27 November 2025) with criteria of oral bioavailability (OB) ≥ 30% and drug-likeliness (DL) ≥ 0.18. These criteria were widely accepted thresholds in network pharmacology for identifying orally bioavailable compounds with drug-like properties [55] and have been validated in numerous TCM network pharmacology studies [56,57]. For *G. elata*, compounds were retrieved from the HERB database (http://herb.ac.cn/v2, access data: 27 November 2025) and further filtered using SwissADME (https://www.swissadme.ch/, access data: 2 December 2025), retaining those with high GI absorption and satisfying all five drug-likeness rules (Lipinski, Ghose, Veber, Egan, and Muegge) [58]. Corresponding targets for these active compounds were obtained from DrugBank (http://www.drugbank.com/, access data: 8 December 2025) and PharmMapper (https://lilab-ecust.cn/pharmmapper/, access data: 8 December 2025), and duplicates were removed to generate a combined target set for the Tianma–Gouteng pair. Disease targets for preeclampsia were collected from GeneCards with relevance score ≥ 1 (https://www.genecards.org/, access data: 20 November 2025), OMIM (http://www.omim.org, access data: 20 November 2025), and DisGeNET with Score > 0.3 (http://www.disgenet.org/, access data: 20 November 2025), also after deduplication. A three-way Venn analysis was performed among “Drug” targets, “Disease” targets, and “Sequencing” targets to identify overlapping candidates. PPI analysis of these intersecting targets was conducted using Cytoscape (version 3.7.2).

### 4.8. Cell Culture

The HTR-8/SVneo cell line was purchased from the American Type Culture Collection. Cells were cultured in RPMI 1640 medium (Gibco, Waltham, MA, USA, 11875093) supplemented with 10% fetal bovine serum (Gibco, Waltham, MA, USA, 10099141) and 1% penicillin–streptomycin (Gibco, Waltham, MA, USA, 15140122). The cells were incubated at 37 °C in a 5% CO_2_ atmosphere and passaged upon reaching 75% confluency.

### 4.9. Trophoblast Organoids Culture

Trophoblast organoids were passaged when they reached a diameter of 200–300 μm sphere. Low-retention 200 μL tips were used for pipetting and Stem Pro Accutase (STEMCELL, Shanghai, China, 07920) was used for digestion. Trophoblast organoids collected from 3–4 wells of a 48-well plate were pooled in 1.5 mL tubes and mechanically disrupted by repetitive pipetting (~400 strokes) with an electronic pipettor. After enzymatic digestion and centrifugation, the organoid pellet was collected and resuspended in Matrigel. Subsequently, 25 μL organoid–Matrigel suspension was pipetted into a 48-well culture plate for passage culturing or 10 μL organoid–Matrigel suspension was pipetted into a 96-well culture plate with a black transparent bottom (Corning, NY, USA, 3904) for IF staining. Finally, 250/100 μL TOM was added to each well for culturing.

### 4.10. Overexpression Experiments

For trophoblast organoids and HTR-8/SVneo cell transfection, overexpressed plasmid was transfected using Lipofectamine 2000 transfection reagent (Invitrogen, Waltham, MA, USA, 11668019) according to the recommended dosage.

The ADRA1A-overexpressed trophoblast organoids and HTR-8/SVneo cells were treated with 10 μM SRH, yohimbine, rhynchophylline, corynoxeine, and beta-sitosterol for validation experiments.

### 4.11. Quantitative Real-Time Polymerase Chain Reaction (RT-qPCR)

Total RNA was extracted using a RNeasy Mini Kit (Qiagen, Hilden, Germany, 74104). Reverse transcription was performed with the TRUEscript RT MasterMix Kit (Aidlab, Beijing, China, PC7002). An SYBR Green PCR Kit (Yeasen, Shanghai, China, 11184ES) was applied in a CFX-Touch™ 96 real-time PCR system (Bio-Rad, Hercules, CA, USA) with the necessary primers (Zhejiang Sunya, Hangzhou, China) for assessing gene expression. The specific primer sequences are listed in Table S5. Relative expression was calculated using the ΔΔ*C*t method and normalized.

### 4.12. Molecular Docking

The crystal structure of the target protein was downloaded from the RCSB Protein Data Bank (PDB), with preference given to high-resolution structures that contain a co-crystallized ligand (PBD ID: 8THK, 2.60 Å, active state). Pre-processing of the protein structure was carried out using AutoDock4.2.6, which involved removing water molecules, separating the ligand from the protein, adding polar hydrogens, and computing Gasteiger charges, after which the structure was saved in pdbqt format. Small-molecule compounds were retrieved from the PubChem database as SDF structures and then converted to pdbqt format using either Open Babel (version 2.4.1) or AutoDock (version 4.2.6), with atomic charges added and rotatable bonds assigned. For defining the docking grid box, the center was set to the co-crystallized ligand when available. In the absence of a ligand, PyMOL (version 3.1.1) was used to predict potential binding sites. The receptor and ligand file paths, grid box center and dimensions, and the number of docking modes were all specified in the configuration file (the docking grid dimensions and coordinates used in our study were as follows: the grid center was set at x = 135.007 Å, y = 138.112 Å, z = 121.737 Å; the number of grid points (npts) was 126 × 126 × 126 along the x, y, and z axes; and the grid spacing was 1.0 Å. Ten docking poses were generated). Upon completion of the docking runs, multiple binding conformations along with their corresponding binding energies were generated. Finally, the intermolecular interactions—including hydrogen bonds, π–π stacking, and hydrophobic contacts—were analyzed using PyMOL and LigPlus (version 2.3.1).

### 4.13. SPR Experiments

The recombinant human ADRA1A was obtained from CUSABIO (Wuhan, China, Catalog No. CSB-CF001385HU). A CM5 sensor chip was used for SPR analysis. The chip surface was activated by injecting a freshly mixed EDC/NHS solution (400 mM/100 mM, 1:1) at 10 μL/min for 420 s. ADRA1A diluted to 20 μg/mL in immobilization buffer was then injected over the sample flow cell (Fc3) at 10 μL/min to achieve a target immobilization level of approximately 8500 RU, leaving the reference cell (Fc1) unmodified. Remaining active sites were blocked with 1 M ethanolamine-HCl at 10 μL/min for 420 s. Six concentrations of isorhynchophylline (25, 12.5, 6.25, 3.12, 1.56, and 0 μM) were injected over both cells at 30 μL/min in ascending order, with 120 s association and 130 s dissociation, using running buffer without regeneration. Sensorgrams were processed by subtracting reference (Fc1) and zero-concentration responses, and the equilibrium dissociation constant (KD) was determined by steady-state fitting using Biacore T200 evaluation software (version 3.2). No additional controls (e.g., solvent correction or unrelated protein) were used.

### 4.14. Safety and Efficacy Evaluation on HTR-8/SVneo

HTR-8/SVneo cells were treated with 3.125, 6.25, 12.5, 25, 50, 100 and 200 μM SRH for safety evaluation. 15 mM L-NAME was used to build L-NAME-induced injury HTR-8/SVneo cell model. Injury HTR-8/SVneo cells were treated with 6.25, 12.5, 25, 50, 100 and 200 μM SRH for efficacy evaluation.

EdU staining kit was purchased from Beyotime Biotechnology (Shanghai, China, C0071S) and was performed according to the manufacturer’s instructions.

### 4.15. Safety Evaluation on Trophoblast Organoids

Trophoblast organoids were categorized into four groups—control, low-dose SRH, medium-dose SRH, high-dose SRH, and control—the latter of which was treated with normal TOM. The remaining three groups were treated with 15, 30, and 60 μM SRH on days 3–4 after passage.

### 4.16. Efficacy Evaluation on Trophoblast Organoids

30 mM L-NAME was used to establish the trophoblast organoids damage model as previously described (patent application CN202511878852.5). L-NAME was added into TOM on day 3 after passage to induce trophoblast organoids damage, and the cultures were left for 2 days. Next, trophoblast organoids were categorized into five groups: control, L-NAME, low-dose SRH, medium-dose SRH, and high-dose SRH. The control group was treated with normal TOM, and the remaining four groups were exposed to 30 mM L-NAME on days 3–4 after passage and subsequently treated with normal TOM, 15, 30, and 60 μM SRH on days 5–6.

### 4.17. Statistical Analysis

All data are expressed as means ± standard error of the mean (mean ± SEM). Sample sizes were predetermined using power analysis (two-tailed *t*-test, Cohen’s d = 1.2, α = 0.05, power = 0.85) and are consistently reported throughout. Statistical significance was assessed using an unpaired *t*-test for comparison between two groups and using one-way analysis of variance (ANOVA) for comparisons among more than two groups. Statistical significance was set at *p* < 0.05. Data analysis was performed using GraphPad Prism 8.0.

## 5. Conclusions

In conclusion, integrated transcriptomic and network analyses prioritized ADRA1A as a candidate target of TGP. SRH showed direct binding to ADRA1A and was associated with partial restoration of Ki67 and HLA-G expression in an L-NAME-induced trophoblast organoid injury model. These findings support a rational for further mechanistic and preclinical investigation of SRH and highlight the utility of trophoblast organoids as a platform for evaluating TGP-derived compounds. However, the present study does not establish that the biological effects of SRH are mediated by ADRA1A, nor does it demonstrate clinical efficacy of TGP or SRH in PE. Future studies involving ADRA1A loss-of-function models and clinical placental samples are warranted to confirm the mechanism and translational potential.

## Figures and Tables

**Table 1:** Binding energy of 5 potential bioactive compounds with ADRA1A.

NO.	Ligand Name	Binding Energy (kcal/mol)
1	Beta-Sitosterol	−7.31 kcal/mol
2	Isorhyncophylline	−6.57 kcal/mol
3	Yohimbine	−6.19 kcal/mol
4	Corynoxeine	−6.09 kcal/mol
5	Rhynchophylline	−5.58 kcal/mol

## Data Availability

The original contributions presented in this study are included in the article/Supplementary Materials. Further inquiries can be directed to the corresponding authors.

## Supplementary Materials

The following supporting information can be downloaded at https://www.mdpi.com/article/10.3390/ph19101540/s1. Table S1: Ingredient and targets of Gouteng; Table S2: Ingredient and targets of Tianma; Table S3: Network-tianmagouteng; Table S4: Type-tianmagouteng; Table S5. Primer sequences in RT-qPCR.

## Abbreviations

The following abbreviations are used in this manuscript.

PE	Preeclampsia
TGP	Tianma–Gouteng pair
ADRA1A	Alpha-1A adrenergic receptor
SRH	Isorhynchophylline
L-NAME	N^G^-nitro-L-arginine methyl ester
sFlt-1	soluble fms-like tyrosine kinase 1
TCM	Traditional Chinese Medicine
GD	Gestational day
TOM	Trophoblast organoid medium
HE	Hematoxylin and eosin
IF	Immunofluorescence
DEGs	Differentially expressed genes
KEGG	Kyoto Encyclopedia of Genes and Genomes
GO	Gene Ontology
SPR	Surface plasmon resonance
